# A Fuzzy Logic Enhanced Environmental Protection Education Model for Policies Decision Support in Green Community Development

**DOI:** 10.1155/2013/250374

**Published:** 2013-12-02

**Authors:** Sung-Lin Hsueh

**Affiliations:** Graduate Institute of Cultural and Creative Design, Tung Fang Design Institute, No. 110 Dongfan Road, Hunei District, Kaohsiung 82941, Taiwan

## Abstract

This study proposes the promotion of environmental protection education among communities as a solution to the serious problems of high energy consumption and carbon emissions around the world. Environmental protection education has direct and lasting influences on everyone in society; therefore, it is helpful in our fight against many serious problems caused by high energy consumption. In this study, the Delphi method and the fuzzy logic theory are used to develop a quantizing assessment model based on qualitative analysis. This model can be used to assess the results and influences of community residents' participation in environmental protection education on green community development. In addition, it can be used to provide references for governing authorities in their decision making of green community development policies.

## 1. Introduction 

The community building in Taiwan has evolved from the simple community improvement and renovation to the holistic development and reengineering of “people, culture, land, scenery and production” in the community. The community building in Taiwan nowadays focuses more on the participation and education of the residents to realize the goal of channeling the consensus and power of the residents into solving difficult problems of their community. Because of its development over the past years, the problem of high-level carbon emissions in Taiwan has become more and more serious. To solve this problem,  environmental protection education has been proactively promoted among communities in Taiwan with professionally trained seed teachers of environmental protection for community residents. In addition, to further enhance the awareness of environmental protection, both teachers and students at the schools of all levels in Taiwan are required to receive education about environment protection. Last but not least, incentives have been offered to encourage participation of community residents in the environment protection courses and activities in their communities. According to O'Neill [1], lessons learned from a national project on education for personal and social responsibility can be adopted across a variety of specific institutional contexts and missions.

In recent years, the Taiwanese government has been proactively promoting a wide variety of policies to encourage energy conservation and carbon emission reduction, such as Green Procurement, Green Buildings [2–4], Environmental Protection [5], Environmental Efficiency [6], Plain Afforestation Policy [7], “Love for the Earth, Let us Go!” community subsidy program, and subsidies for purchases of green household appliances and solar power devices. However, despite the large amounts of governmental budgets invested in the above-mentioned policies and subsidies each year, the problem of high carbon emissions from the coal-fired power plants, petrochemical industry, and large population of automobiles and motorcycles has shown no sign of significant improvement. Probably the relatively effective solution to solve the problem of high carbon emissions in Taiwan in the long run is promoting the awareness of environmental protection and low-carbon lifestyle through environmental protection education. Low-carbon lifestyle plays a potential role in directly deciding the household livelihood and economic development in Taiwan in the future. To achieve low-carbon lifestyle among people in Taiwan, it requires recognition and cooperation from everyone on this island.

Much of society's use of energy is to satisfy heating and cooling preferences [8]. The greenhouse effect and extreme weather of the Earth are caused by heat and carbon emissions from human activities [9–11]. For most energy users in society, imposition of energy taxes and carbon emission taxes on them does not lead to effective, lasting, and significant results. Worsening climate-related disasters around the world have seriously threatened not only the biodiversity of species on the Earth but also lives and properties of all humanity. Climate change is a major current affair [12]. According to Böhm and Pfister [13], environmental risks pose a serious problem to individual and societal decision-making. Therefore, it is of outmost importance for everyone around the world to address environmental risks. There are already many attempts by scientists and researchers to address environmental risks, such as the research by Brondi et al. [14] in addressing environmental concerns from an applied social psychological perspective.

Dounis and Caraiscos [15] pointed out that the problem of energy conservation is a multidimensional one. The high carbon emissions and global warming around the globe have brought more unpredictable climate disasters such as droughts, floods, and rising sea levels. However, these disasters are all regional problems. The severity of these disasters is not felt by most people outside the areas where these disasters occur [16]. In addition, everyone around the world has different requirements for energy use while each country has its own consideration of industrial development and economic interests. It is difficult to reach consensus among different individuals and countries on reduction of energy consumption. Moreover, measures to curb carbon emissions like carbon tax or carbon trade are likely to be perceived as kinds of economic games to the benefit of certain entities. Therefore, it is understandable that the promotion of these measures has failed to reach significant results.

By exploring the promotion of environmental protection education in the communities in Taiwan, this study is intended to develop an effective model that can help a community to assess its green community development through environmental protection education. In the first step of building this model in this study, the collective decision-making function of the Delphi method is used by consulting a group of governmental, industrial, and academic experts to confirm the courses and their contents of the environmental protection education for a community and their suitability for community residents. Then the quantization methods of the fuzzy logic theory are used to convert each course into a quantifiable criterion. By doing so, the environmental protection awareness among the community residents before and after taking the courses can be measured against the criteria to quantize the influences of the environmental protection education on the community as a whole. In addition, the progress of the green community development can be easily quantized by this assessment model to provide policy-making references for governing authorities. In addition, this assessment model developed in this study is highly adaptive and easy to modify and adopt in different scenarios for future research.

## 2. Model Overview

**Figure 1:**
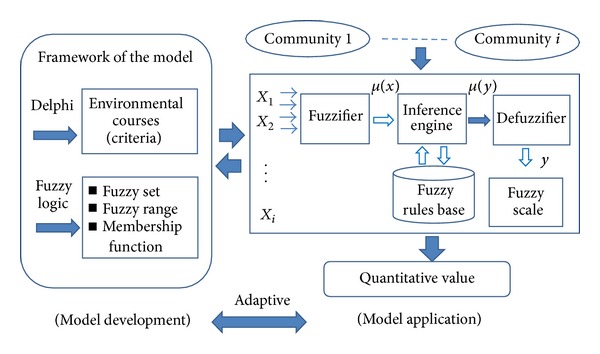
The framework of the evaluation model.

This section mainly explains (1) steps of model development and (2) steps of model application in this study. As illustrated in Figure 1, there are three steps of model development in this study.

 Step 1: through the collective decision-making approach of the Delphi method, courses related to green community development are decided. These courses also serve as criteria to assess the recognition of environmental protection among community residents.
 Step 2: with the assistance from the Delphi experts, the fuzzy set, fuzzy range, and membership function are decided for the fuzzy logic quantizing.
 Step 3: build the inference rule base and complete the fuzzy logic inference system (FLIS).

There are also three steps of model application in this study.

 Step 1: define the assessment targets, resident(s) or community/communities (community of one individual or more).
 Step 2: after the assessment targets are defined (as *X*_1_, *X*_2_,…*X*_*i*_), they are sent to the fuzzifier and converted into corresponding semantic values in accordance with the inference rule base. Then they are sent to the defuzzifier and converted into quantized output values.
 Step 3: the quantized output values can be used to make comparisons and provide references for policy-making judgments.

## 3. Delphi Method and Fuzzy Theory

First developed and used by the RAND Corporation in the US in 1946, the Delphi method is a tool to facilitate management and to predict the future. It can be used to form high-quality decision making based on opinions and judgments collected from each individual member. Its practical values are proven in many aspects of the complicated modern society. The Delphi method is a technology for collective decision making. Having been widely applied in exploring different kinds of issues, the method can be used in building an ideal future [17] or in research activities such as the development of The Working Competency Items for Energy Technology [18], research on academic libraries [19], and graduate research [20]. The Delphi method refers to industrial experts of related specialized fields, experts of government competent authorities, and scholars with practical experience to provide the latest knowledge meeting existing circumstances and future development trends. The Delphi method was developed by the RAND Corporation in the US back in the 1950s. It is a method that assists management as a prediction tool, especially for collecting the opinions and judgments of individual members to form high quality decisions and has been widely used in complex societies [21, 22].

**Figure 2:**
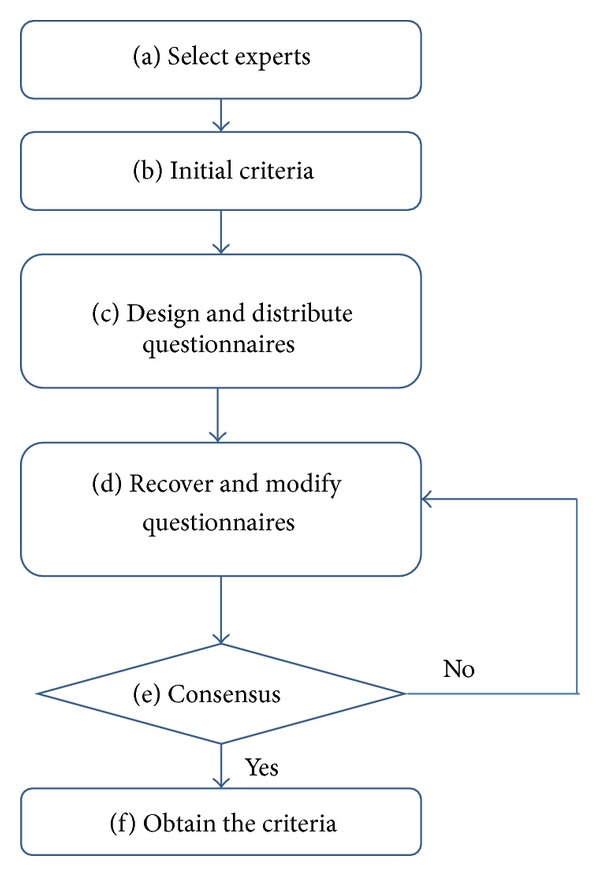
Delphi method operation flowchart.

The Delphi method is composed of the following steps: (a) select experts; (b) obtain initial assessment factors from previous studies; (c) design and distribute questionnaires; (d) recover and modify questionnaires; (e) if assessment factors do not reach a consensus, return to Step (d); (f) obtain the criteria required for this study [2].  Figure 2 shows a flowchart of the Delphi method process.

The fuzzy theory was developed by Professor L. A. Zadeh of the University of California Berkeley in 1965 [21, 22]. It is the best quantization tool for processing fuzzy phenomena and fuzzy meaning. The fuzzy theory is based on fuzzy sets that express and quantize indefinable fuzzy concepts; it has a good effect on processing the fuzzy phenomena of particular expressions of human languages. The fuzzy set theory extends the set theory of dichotomy (set value is 0 or 1) of the traditional mathematical theory into infinitely continuous set values (set value is 0~1).

## 4. Development of the Assessment Model

The assessment model developed in this study is a quantization model based on qualitative analysis. The model development starts with consulting the Delphi experts in this study to design and confirm the environmental protection education that all the experts agree to be suitable for the assessed community and helpful for its development as a green community. The course contents of the environmental protection education are intended to promote awareness among community residents about the importance of environmental protection and help the community to achieve sustainable development. Therefore, each of the courses in the environmental protection education also serves as a criterion to assess improvement brought by the education. Then, the fuzzy scale of each criterion is defined to complete the fuzzy logic inference system (FLIS). The fuzzy logic theory is a scientific tool for quantization. It can be used to convert and measure the human semantic function while it can tolerate uncertainty and fuzziness.

### 4.1. Selection of Initial Criteria

A community is the most important collective body in social activities and also a fundamental unit in urban development. It is easier to promote consensus among community members through community organizations. Green community development requires concrete actions of the community residents to protect the environment and conserve energy. Since issues about the environment and energy cover a wide range of fields, the implementation of environmental protection education and sustainable energy education can be a very difficult and long-term task. According to Hurtado and Hunte [23], sustainable energy education must be structured in an interdisciplinary manner whereby engineering modules are complemented with legal, social, economic, managerial, and environmental coursework.

The preliminary criteria of environmental protection education in this study obtained from the literature review are community energy-saving solar-assisted heat [24], economical use of natural energy resources [25], resource recovery and reuse, energy-saving materials [26], planning and design of energy-efficient equipment and energy-saving construction [27], urban greening [27], sustainable energy development [23], and energy and environmental efficiency [6].

There are totally 16 Delphi experts in this study—four experts of industrial circles, six resident representatives of green community organizations, three experts of governmental authorities, and three scholars with practical experiences. Since this study aims to promote green community development in Taiwan, the Delphi experts suggested that the environmental protection education in this study should focus on community residents and incorporate more participatory teaching strategies [28]. After an 8-month period of Delphi process in this study, the course contents and teaching methods of environmental protection education beneficial for green community development are confirmed, such as participation of the residents in action performance, watching environmental protection promotion films, enhancing knowledge about energy-saving materials and equipment among the residents, community greening, and problem based learning [29]. The criteria applicable to this evaluation model as shown in Table 1 are obtained with the assistance of the Delphi experts. Each criterion in the table is agreed upon by all the experts.

### 4.2. Definition of Membership Function, Fuzzy Set, and Fuzzy Range

The fuzzy logic theory usually uses the Mamdani or Sugeno systems for modeling, where the quantized output values of the Mamdani system are continuous changes, and the quantized output values of the Sugeno system are discrete changes. This study used the Mamdani system for modeling in order to present the continuous output change values of the model. A membership function characterizes a fuzzy linguistic term by giving its support value or degree of membership. The membership value varies from 0 to 1, representing none to full membership. Common membership functions include triangular- and bell-shaped functions [30]. The membership functions in this study are bell-shaped functions for their curves are smoother and, therefore, more suitable for the continuous output changes of the Mamdani system.

Table 2 lists the number of fuzzy sets of each criterion: five (Very good, Good, Normal, Poor, and Very poor) in performances activities, three (Good, Normal, and Poor) in energy-saving public facility, three (Good, Normal, and Poor) in energy-saving family; and three (Good, Normal, and Poor) in problem based learning. The fuzzy sets of the four criteria can be combined to form 135 scenarios.

Fuzzy logic can tolerate different measure units and fuzzy ranges. It is a tool which uses computer algorithms to process tasks difficult for human processing. The definition of a fuzzy range can vary from 0 to 1, just like that of a membership value. As for those semantic meanings easy to understand or those measure units frequently used such as the performance activities in this study, their fuzzy ranges can be defined by their quality levels perceived by the audience or the participants, such as 90 points for “Very good,” 80–89 points for “Good,” 70–79 points for “Normal,” 60–69 points for “Poor,” and less than 59 points for “Very poor.” The Delphi experts suggested that the fuzzy range of performance activities should be defined by the participation rates of the community residents for the more community residents participate in the activities, the better results of interactive learning can be achieved. Therefore, the fuzzy range of performances activities is defined as follows: at the range from 10% to 40% with 10% or lower meaning “Very poor” while 40% and higher meaning “Very good.” As for the criteria of public facility energy-saving and family energy-saving, the Delphi experts suggested that, since environmental protection education is supposed to be able to promote lower energy consumption in the community and the household, the fuzzy range definitions of these two criteria are both set from 0% to 30% of energy conservation (with higher percentage meaning better energy conservation). As for the problem based learning, the Delphi experts suggested that if a community can develop over ten cases each month that can provide references for other communities in their problem-solving learning and improvement, it indicates that the community is on the right track of sustainable community development. However, the experts also indicated that the number of such cases can be adjusted based on the actual conditions of each community. Therefore, the fuzzy range definition of problem based learning is set from 0 to 10 in this study.

The definitions of the evaluation scales of the four criteria, the upper and lower limits of each scale, and the definition of output quantized scale range are as shown in Table 3. The measurement scale defined in fuzzy logic is a man-made fuzzy scale; for example, when the criterion of energy-saving family common understanding is energy-saving more than 30%, it means “Very good,” 20% means “Good,” 15% means “Good or Normal,” 10% means “Normal,” 5% means “Poor,” and below 5% means “Very poor.” The membership function in the fuzzy logic scale defines good or normal, and then the fuzzy logic inference system is used for defuzzification to complete the output results of quantized values.

### 4.3. Establish Fuzzy Logic Inference System

The establishment process of FLIS requires (1) inputting the selected criterion and the definition of fuzzy sets, (2) inputting the definition of the fuzzy sets of output values, (3) establishing the rule base of IF-THEN, (4) considering membership functions, and (5) obtaining the corresponding quantitative output value (figure or ratio) after FLIS defuzzification [21].

The fuzzy logic inference system (FLIS) makes inferences according to the IF-THEN Rules and defuzzifies the results, which are used to determine the output quantized values. As shown in Table 2, the four criteria can form 5∗3∗3∗3 (135) different input scenarios. The IF-THEN Rules of the fuzzy logic in the overall FLIS act as a human brain; when the IF-THEN Rules of a FLIS are complete, the FLIS has the function of calculation. When the decision maker gives each criterion an input scenario, the FLIS can automatically determine the quantized effect evaluation value.

## 5. Output Quantized Value

**Figure 3:**
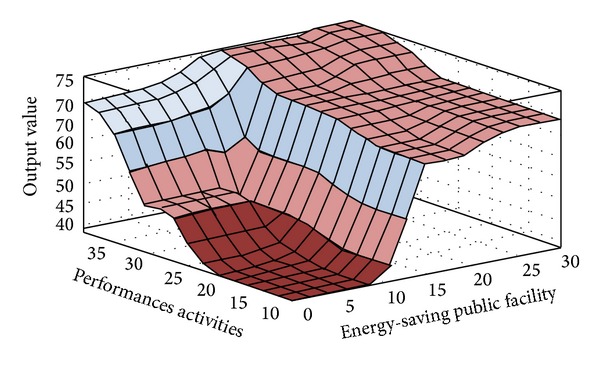
Input and output 3D mapping (performances activities and energy-saving public Facility).

**Figure 4:**
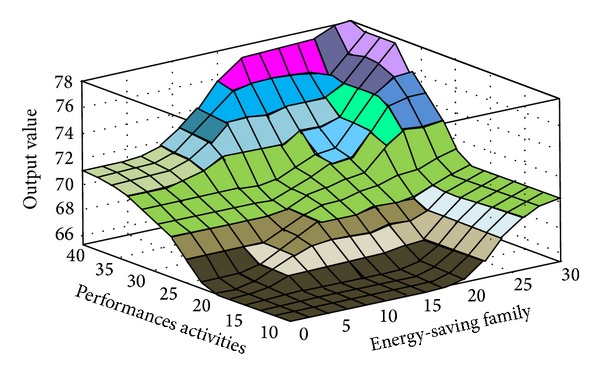
Input and output 3D mapping (performances activities and energy-saving family).

**Figure 5:**
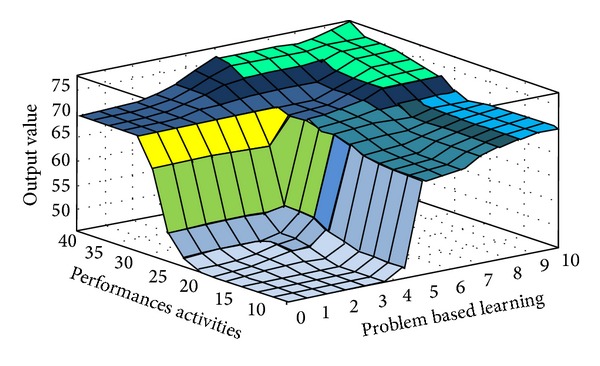
Input and output 3D mapping (performances activities and problem-based learning).

**Figure 6:**
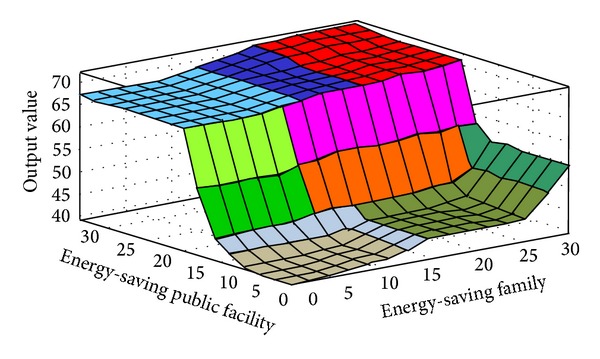
Input and output 3D mapping (energy-saving public facility and energy-saving family).

**Figure 7:**
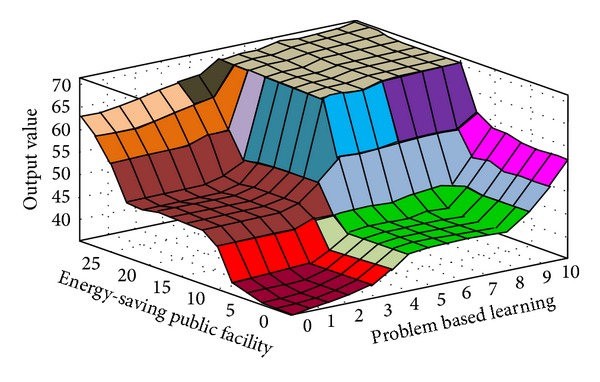
Input and output 3D mapping (energy-saving public facility and problem based learning).

**Figure 8:**
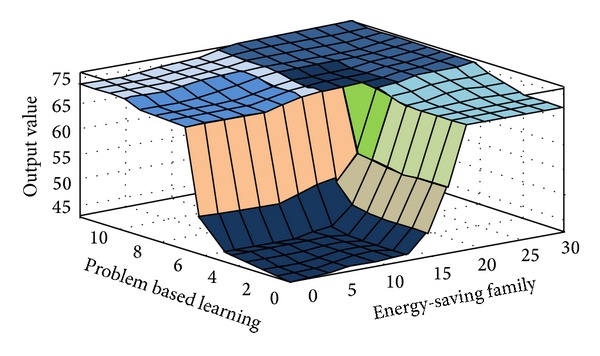
Input and output 3D mapping (problem based learning and energy-saving family).

The fuzzy quantized values of the various evaluation factors converted by FLIS are the criteria for evaluating “Influences of Environmental Protection Education on Green Community Development.” Higher scores obtained from FLIS mean higher value. Figures 3, 4, 5, 6, 7, and 8 show the quantized output 3D evaluation relations of FLIS in various scenarios. The relations among various evaluation factors can be observed from the 3D evaluation relation chart.  Table 4 shows that the quantized score result of the best scenario is 84.4 and the quantized score result of the worst scenario is 28.7.

## 6. Case Study

The community studied in this research is the Dahu Community in Kaohsiung city of Taiwan. From 2011 to 2012, the Dahu Community received the governmental subsidy for the “Environmental Protection and Green Energy Development Program” (Case 1), which is part of the government's Plain Afforestation Policy for Carbon Sequestering [7]. The model developed in this study is first used to assess the participation of the Dahu Community residents in the program's environmental protection education during the Delphi process that started from August 1st, 2011. The semantic evaluation results of each criterion after the Delphi process of Case 1 are listed as follows:

- performances activities—Poor,
- energy-saving public facility—Low,
- energy-saving family—Low,
- problem Based Learning—Common.

As shown in Table 5, the output quantized score of Case 1 is 54.2. From 2012 to 2013, the Dahu Community received another governmental subsidy for the “Love for the Earth, Let us Go! Program” (Case 2), which is part of the government's Green Community Development Policy. Compared with the previous program, the program in Case 2 focuses more on environmental protection education for community residences and their participation. The model developed in this study is used again to assess each criterion during the Delphi process that started from August 25th, 2013. The assessment results of Case 2 are listed as follows:

- performances activities—Good,
- energy-saving public facility—Moderate,
- energy-saving family—Moderate,
- problem based learning—Hot.

As shown in Figure 2, the output quantized score of Case 2 is 74.3. The quantized comparison between Cases 1 and 2 in this study demonstrates that green community development policies combined with environmental protection education have better development results. In addition, such policies can win more participation from community residents and have higher levels of problem based learning. These findings demonstrate the positive influences of environmental protection education on community development. However, in Case 2, the criterion of public facility energy-saving or family energy-saving does not show significant improvement from Case 1. It is probably because improvement or installation of energy-saving facilities or device requires extra expenditures for both the household and the community. According to the case analysis, when any scenario is entered in the FLIS, the system will automatically determine the quantized output value; the FLIS can help evaluators to rapidly compare different evaluated communities.

## 7. Conclusions

A community is an important and basic unit in urban development and social activities. A community with consensus among its members is more likely to develop its own characteristics. Community building policies that focus on participation of community residents can help to promote consensus of community residents. In addition, from the comparison of Cases 1 and 2 in this study, it is found that green community policies combined with environmental protection education are more likely to be accepted by most community residents, which can consequently help to ensure better results of the policies. However, it is also found in this study that, even though most of the community residents are aware of the importance of green community development through environmental protection education, the lack of funds for the improvement or installation of energy-saving facilities and devices in the community and the household can be a serious problem that can hinder the green community development. Fortunately, this problem can be solved through governmental policies such as subsidies or low-interest-rate loans for the communities. Lastly, the assessment model developed in this study is highly objective and adaptive. It can be used by not only the community to assess their development progress but also the government to find references for its policy making.

## Figures and Tables

**Table 1:** Criteria jointly agreed by experts.

Each criterion	Content
Performances activities	Participation of the community residents in action performance, experience/suggestion sharing, watching environmental protection promotion films, and other methods of interactive learning/teaching.

Energy-saving public facility	Education about energy-saving public facilities in the community, such as energy efficient design, solar electric systems, use of energy-saving materials, automatic street lamp monitoring, water, energy saving devices, and Plain Afforestation Policy (Carbon Sequestering).

Energy-saving family	Education about what a family can do to achieve energy conservation and environmental protection, such as family planting, economical use of natural energy resources, use of water-saving/energy-saving household appliances, resource recovery, and reduction of household waste production.

Problem based learning	According to the Delphi experts in this study, environmental protection education is cross-disciplinary; therefore, an environmental protection organization must be established in the community to access and integrate the cross-disciplinary specialty of all its community residents in detecting and solving the related problems in the community. Through their long-term participation and problem-solving practices, all the residents can learn the latest cross-disciplinary knowledge about environmental protection.

**Table 2:** Numbers of fuzzy sets in each criterion and total scenarios.

Each criterion	Fuzzy set
Performances activities	5
Energy-saving public facility	3
Energy-saving family	3
Problem based learning	3

Scenario	5∗3∗3∗3 = 135

**Table 3:** Fuzzy set range definition of input and output criteria of community energy-saving.

Input criteria			Output	
Name of each criterion	Value range	Fuzzy set	Name	Fuzzy set
Performances activities	10%–40%	Very good	Quantized value	Very good (above 90 points) Good (above 75 points) Normal (above 60 points) Poor (about 50 points) Very poor (below 30 points) (0–100%)
		Good		
		Normal		
		Poor		
		Very poor		
Energy-saving public facility	0%–30%	High		
		Moderate		
		Low		
Energy-saving family	0%–30%	High		
		Moderate		
		Low		
Problem based learning	0–10	Hot		
		Common		
		Blue		

**Table 4:** Quantized output of the best and the worst.

Scenario	Optimal	Worst
Performances activities	Very good	Very poor
Energy-saving public facility	High	Low
Energy-saving family	High	Low
Problem based learning	Hot	Blue

Output value	84.4	28.7

**Table 5:** Output values of Case 1 and Case 2 from FLIS calculation.

Scenario	Optimal	Worst
Performances activities	Poor	Good
Energy-saving public facility	Low	Moderate
Energy-saving family	Low	Moderate
Problem based learning	Common	Hot

Output value	54.2	74.3
